# A Cluster Head Selection Algorithm for Extending Last Node Lifetime in Wireless Sensor Networks

**DOI:** 10.3390/s25113466

**Published:** 2025-05-30

**Authors:** Marcin Lewandowski, Bartłomiej Płaczek

**Affiliations:** Institute of Computer Science, University of Silesia, Będzińska 39, 41-200 Sosnowiec, Poland; marcin.lewandowski@us.edu.pl

**Keywords:** wireless sensor network, lifetime of sensor network, cluster head rotation, transmission reduction, internet of things

## Abstract

This paper introduces a new cluster head selection algorithm for wireless sensor networks (WSNs) to maximize the time until the last sensor node depletes its energy. The algorithm is based on a formal analysis in which network lifetime is modeled as a function of node energy consumption. In contrast to existing energy-balancing strategies, this analytical foundation leads to a distinctive selection rule that prioritizes the node with the highest transmission probability and the lowest initial energy as the initial cluster head. The algorithm employs distributed per-cluster computation, enabling scalability without increasing complexity relative to network size. Unlike traditional approaches that rotate cluster heads based on time or equal energy use, our method adapts to heterogeneous energy consumption patterns and enforces a cluster head rotation order that maximizes the lifetime of the final active node. To validate the effectiveness of the proposed approach, we implement it on a real-world LoRaWAN-based sensor network prototype. Experimental results demonstrate that our method significantly extends the lifetime of the last active node compared to representative state-of-the-art algorithms. This research provides a practical and robust solution for energy-efficient WSN operation in real deployment scenarios by considering realistic and application-driven communication behavior along with hardware-level energy consumption.

## 1. Introduction

Wireless sensor networks (WSNs) are essential components of the IoT that enable data collection through multiple sensor nodes located in different areas. Data collected by WSNs can be processed for various purposes, including process monitoring, decision-making, event recognition, control, and others [1]. The design and development of WSNs encounters a range of specific constraints that do not exist in traditional computer networks. Sensor nodes are usually powered by an embedded battery with limited capacity. In many cases, replacing or recharging batteries in sensor nodes is impractical [2] and the sensor nodes with discharged batteries are no longer used. For these reasons, operations performed within a WSN should consider the need for efficient energy use and ensure sufficiently long operational times for sensor nodes. Addressing these constraints requires the development of dedicated data transmission algorithms to extend the lifetime of WSNs. While the need for energy conservation applies to all aspects of sensor node operation, data transmission has the most significant impact on energy consumption [3,4,5]. Previous works have suggested that the energy consumption of the data transmission module is significantly higher than the energy consumption of the microcontroller that manages sensor node operations [6].

**Figure 1:**
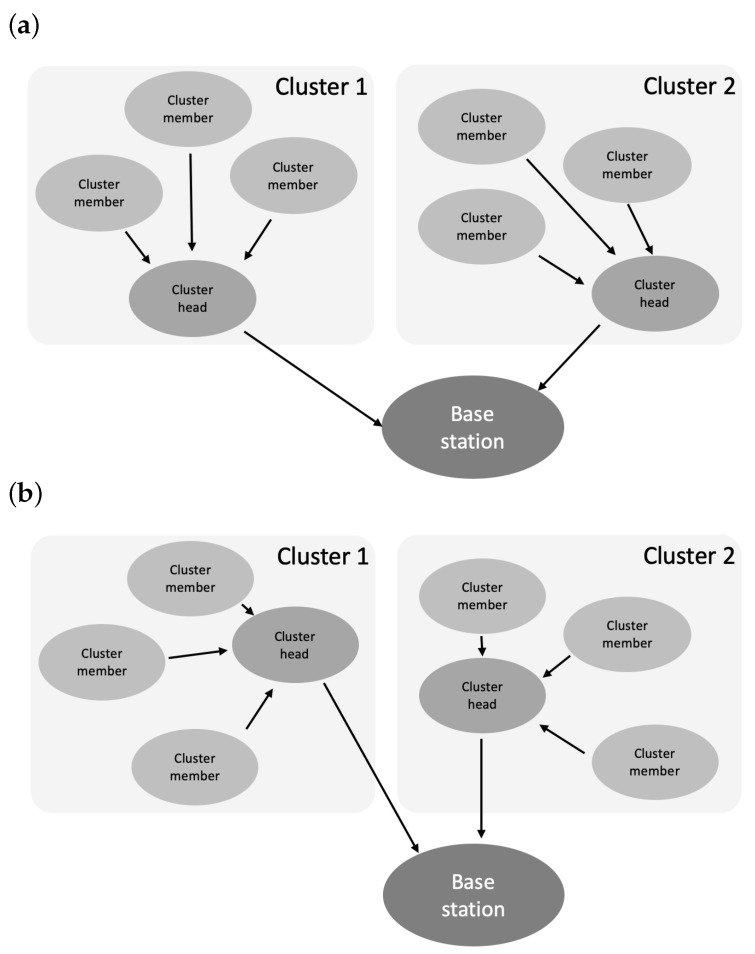
Operation of the sensor network: (**a**) in the n-th round and (**b**) in the (n + 1)-th round.

To ensure the possibility of effective data acquisition from many sensor nodes and reduce the number of transmissions, WSNs can be clustered, as shown in Figure 1. Clustering of WSNs was introduced to achieve effective energy and transmission management by grouping network nodes into clusters in which one node acts as the cluster head [7,8]. The cluster head node is responsible for coordinating communication with both its cluster members and the base station. Due to the tasks it performs, the cluster head node consumes energy resources faster than the remaining nodes (cluster members). Therefore, the role of the cluster head must be transferred (rotated) between those nodes belonging to the cluster [9,10,11]. The concept of cluster head rotation is illustrated in Figure 1. The network consists of two clusters, each containing four sensor nodes and a single base station located externally. In Figure 1a, which represents the n-th round, one sensor node in each cluster is designated as the cluster head, while the remaining nodes act as cluster members, sending their data to the respective cluster head for aggregation and subsequent transmission to the base station. In Figure 1b, corresponding to the (n + 1)-th round, the roles have rotated, with new cluster heads selected in each cluster. In this paper, we propose a new algorithm for selecting the cluster head node. Our proposed algorithm effectively extends the time until the last node in the WSN cluster dies, i.e., depletes its energy resources.

Currently, available algorithms for selecting the cluster head and rotating the role of cluster head node focus mainly on extending the time until the available energy in one of the sensor nodes is discharged (i.e., until the first node dies) [12,13,14]. These algorithms usually delay the time until the first node in a WSN cluster dies by evenly distributing the energy consumption. Such approaches are ineffective when the aim is to prolong the time until all nodes deplete their energy resources (i.e., until the last node dies). Therefore, we introduce a new algorithm to prolong the last node lifetime. The considered time to death of the last node is essential in applications where it is necessary to obtain data on the monitored process for as long as possible, even if the death of subsequent nodes limits the scope of these data [15]. For instance, extending the time to death of the last node is a more meaningful metric for applications where WSN data serve as input for machine learning models that can handle missing values. By ensuring that at least one node remains active for as long as possible, the system can continue to provide valuable data for decision-making, classification, or prediction.

Another motivation for our work is that available cluster head rotation algorithms do not allow for fully efficient use of energy resources when nodes in a WSN autonomously decide on the need for transmission based on current sensor readings. In modern energy-aware sensor nodes, sensor readings are analyzed locally and transmission only occurs if the data are useful for a given application. Thus, data transmission is performed irregularly (with different probabilities) and nodes use their energy resources at different rates. The new method presented in this paper takes into account the transmission probabilities of individual sensor nodes when selecting the cluster head, leading to extended network lifetime.

The new challenge we address in this paper is the design of a cluster head rotation method that explicitly considers the non-uniform transmission probabilities of nodes. This approach more accurately reflects real-world deployments that involve event-driven or application-aware sensing. The proposed method aims to maximize the lifetime of the last node by integrating these heterogeneous transmission behaviors into the cluster head selection process.

The main contributions of this work are summarized as follows:

- We show that the last node lifetime in a WSN cluster strongly depends on the order in which the sensor nodes take on the role of cluster head.
- We demonstrate that the most effective order can be found by taking into account the initial energy levels and transmission probabilities of sensor nodes. We propose an algorithm for selecting the cluster head which ensures a longer time to death of the last node.
- We provide results of experimental lifetime evaluations using a real-world WSN prototype based on LoRaWAN technology, and compare the results achieved by our proposed algorithm to those achieved by state-of-the-art solutions.

The rest of this paper is structured as follows. Section 2 includes a review of the current state of science and technology in methods for extending the lifetime of sensor networks, particularly through the selection of cluster head nodes. Based on the conducted literature review, we identify the advantages, limitations, and drawbacks of the available methods. Section 3 includes a presentation of the proposed method which enables the extension of the lifetime of the sensor network. Section 4 presents a model for determining the lifetime of a wireless sensor network. This model is then used to conduct a qualitative experimental evaluation of cluster head node selection methods. The key elements of the model are the data transmission algorithms implemented by individual nodes and the parameters describing the energy consumed by the sensor nodes at different stages of network operation. Section 5 presents the conducted experiments and results confirming the usefulness of the developed solutions. It should be noted that the proposed algorithms were implemented in real sensor network nodes. This is particularly important for evaluating the proposed algorithms, as it confirms their feasibility for practical implementation. Finally, a summary of the obtained results, conclusions, and future research directions on methods for extending the lifetime of sensor networks is provided in Section 6.

## 2. Related Works

Various cluster head selection and rotation approaches have been proposed in the literature to extend the lifetime of WSNs. These approaches are based on different definitions of network lifetime and take into account the specific applications and topologies of WSNs. In this section, we characterize the main categories of the existing approaches and discuss our proposed cluster head rotation scheme in this context.

The simplest method of organizing data exchange in WSNs is direct transmission. This method assumes that all sensor nodes play the same role and that their task is to send collected data directly to the base station [16]. With such assumptions, there is no division of the network into clusters, and consequently no distinction in the role of the cluster head. It should be noted that direct transmission of all collected data to the base station results in high energy consumption and short sensor node lifetimes. In addition, the direct transmission method has low scalability, as the base station in this approach must receive and process large portions of data from all sensor nodes. If the base station’s capacity is exceeded, data from the sensor nodes may be lost. The complexity of this method is low and its hardware implementation is straightforward; however, the disadvantages mentioned above mean that the direct method is very rarely used in practice.

The limitations of direct transmission have led to the development of more advanced data collection methods taking into account the division of nodes in the sensor network into clusters. These methods reduce the number of data transmissions to the base station and lower the energy consumption of the sensor nodes. Nodes belonging to a cluster transmit data to the cluster head. Then, the cluster head aggregates the received data and for transmission to the base station. As mentioned in the Introduction, the role of the cluster head must be transferred between nodes in order to balance the energy consumption and avoid a situation in which a node acting as the cluster head for a long time would quickly deplete its energy resources.

The existing algorithms for rotating the role of cluster head can be divided into two categories. The first category includes time-based algorithms, in which sensor nodes act as cluster heads for a predefined period, while the second category consists of algorithms considering the level of consumed or available energy in a node. An example is an algorithm which changes the role after a predefined energy consumption threshold is exceeded [17,18].

A distributed algorithm for organizing sensor nodes into clusters has been implemented in the hierarchical routing protocol known as the Low-Energy Adaptive Clustering Hierarchy (LEACH) [19]. In this approach, the role of cluster head is rotated among the nodes, with a new cluster head randomly selected after a predefined period. The responsibilities of the cluster head include creating a Time Division Multiple Access (TDMA) schedule that allocates access to the transmission medium for multiple cluster members. Each cluster member transmits data during the time slots assigned by the cluster head. The cluster head collects these data for forwarding to the base station. The operation of the LEACH protocol is divided into rounds, each consisting of two phases: the setup phase and the transmission phase. During the setup phase, the cluster head is changed and data containing information about the roles of individual nodes are disseminated. In the transmission phase, actual data transmission begins, which means that the sensor nodes send their data to the cluster head according to their assigned time slots. LEACH enables cluster heads to perform local data aggregation within each cluster, reducing the amount of data transmitted to the base station [20,21].

There are several well-known variants of the LEACH protocol, each designed to address specific limitations of the original approach by modifying the cluster head selection strategy, clustering mechanism, or communication scheme. These extensions aim to improve energy efficiency, load balancing, or adaptability to network conditions such as node mobility or non-uniform energy distribution. Table 1 summarizes the key characteristics of selected LEACH-based protocols, including LEACH, LEACH-C [22], LEACH-D [23], and LEACH-F [24], based on their design principles and operational differences.

In the HEED (Hybrid Energy-Efficient Distributed Clustering) protocol [25], new cluster heads are also selected after a predetermined duration of one round. This approach is similar to the operation of the LEACH protocol described earlier; however, in the HEED protocol, selection of the cluster head takes into account the remaining energy of each sensor node. The probability of a given sensor node being selected as the cluster head is proportional to the amount of energy available at that node. A distributed procedure is used to select the head of the cluster, which is based solely on local information about neighboring nodes. Nevertheless, this process incurs additional energy costs during data exchange between nodes.

Another method of rotating the role of the cluster head among the sensor nodes is ANTCLUST [26]. As in previous approaches, this method selects the cluster head at the beginning of each round. In order to improve the network lifetime, the ANTCLUST algorithm selects a new cluster head depending on the battery charge level and the distance to neighboring sensor nodes. A specific feature of this method is the use of a clustering algorithm based on a nature-inspired ant colony model.

An alternative approach is the Stable Election Protocol (SEP) [27], which was developed for heterogeneous sensor networks in which the sensor nodes have different initial energy levels. Similar to the LEACH protocol, the SEP protocol randomly selects new cluster heads at the beginning of each round. The advantage of this approach is that sensor nodes with higher available energy are more frequently chosen as cluster heads compared to those with lower energy resources. This method prolongs the time until the first node dies.

A variation of the protocol discussed above is Prolong-SEP (P-SEP) [28], which was developed to extend the lifespan of sensor networks that operate based on fog computing. This algorithm aims to balance the energy consumption of all nodes in order to maximize the lifetime of the network. Nodes with an energy level above a specified threshold are nominated as candidates for the role of cluster head, then the cluster head is randomly selected from among these candidates while considering their distance to other nodes. It is important to note that in both the SEP and P-SEP, the selection of the cluster head is part of a complex clustering procedure; consequently, SEP and P-SEP may be inefficient when frequent changes in node roles within the network are necessary.

Another solution is the Hamilton Energy-Efficient Routing (HEER) protocol [29]. The HEER protocol utilizes a greedy algorithm to establish a Hamiltonian path among sensor nodes within each cluster. This path dictates the organization of data transmissions. The sensor nodes positioned along the path take turns serving as the cluster head. Similar to the other methods mentioned above, the role of cluster head is rotated after a predetermined amount of time.

The energy consumption of the sensor nodes is taken into account in the Energy-Distance Aware Clustering method (EDAC) [30]. In this method, the cluster head is selected based on two key parameters: the remaining energy in the node, and the energy used to send data from other nodes to the potential cluster head. Based on these two parameters, a metric is developed to facilitate uniform energy consumption among sensor nodes. This metric also aids in determining the optimal timing for changing the cluster head.

In the case of the Energy-Distance Aware Clustering Scheme (E-DACS) for wireless sensor networks [31], the rotation of node roles is determined by considering the remaining energy of each node, the mutual distances between nodes, and the distance to the base station. A new cluster head is selected periodically when the energy level of the current cluster head falls below that of any other sensor node within the cluster.

Another algorithm for transferring the role of the cluster head is Energy-Driven Cluster Head Rotation (EDCR) [26]. This algorithm operates based on the relative levels of remaining energy in the nodes within a given cluster. The sensor node with the highest energy level is designated as the cluster head. A dynamically determined threshold triggers the role rotation procedure. The threshold is calculated using the following formula: P·EC, where 0<P<1 is a constant and EC represents the remaining energy in the node. The EC parameter is measured at the moment when the node is selected as the cluster head. In this method, the selection of a new cluster head occurs when the remaining energy of the current cluster head falls below the threshold value. According to this algorithm, the lower the available energy in the nodes, the more frequently the cluster head role is switched. This strategy aims to balance energy consumption among sensor nodes.

The existing methods described above all have significant disadvantages. The primary issue is the assumption that all sensor nodes within a cluster transmit their data regularly at equal time intervals. However, it is important to note that each node may transmit data at different frequencies during specific network operation steps, for instance, to eliminate redundant or irrelevant information. This variability results in dynamic changes in energy consumption among the nodes, which can differ significantly; consequently, existing methods are ineffective in extending the network lifespan, as they do not account for the differences in energy consumption among individual nodes [11,32].

Our proposed method for rotating the role of the cluster head considers the probability of data transmission by specific sensor nodes. By taking into account the transmission probabilities of individual nodes, our algorithm achieves more accurate estimation of energy consumption and provides improved management of the energy resources available within the network.

Another disadvantage of current methods is that they are primarily designed to prolong the time to the death of the first sensor node; consequently, new approaches are needed for applications in which the goal is to extend the time to the death of the last node in the WSN.

In addition, the methods in the literature do not account for parameters that describe energy consumption, which varies depending on the radio transmission technology employed in a specific WSN. Our proposed method incorporates parameters from the energy consumption model that can be estimated based on the known characteristics of hardware solutions, including radio modules. These parameters enable more accurate prediction of energy consumption and node lifetime, which facilitates more appropriate cluster head selection and extends the overall network lifetime.

## 3. Proposed Method

In this paper, we examine the lifetime of a WSN, defined as the duration until the energy of all nodes in the network is depleted. Formally, the lifetime of the network (LT) can be expressed as follows:

(1) $$LT=\max\{t:\max_{i\in I}\;\left\{e_{i}\left(t\right)\right\}>\epsilon \}$$

where $e_{i}\left(t\right)$ denotes the available energy for sensor node *i* at time step *t*, *I* is the set of identifiers for all nodes in the WSN, and ϵ stands for the minimum energy required by a sensor node to perform its operations during a single time step.

**Figure 2:**
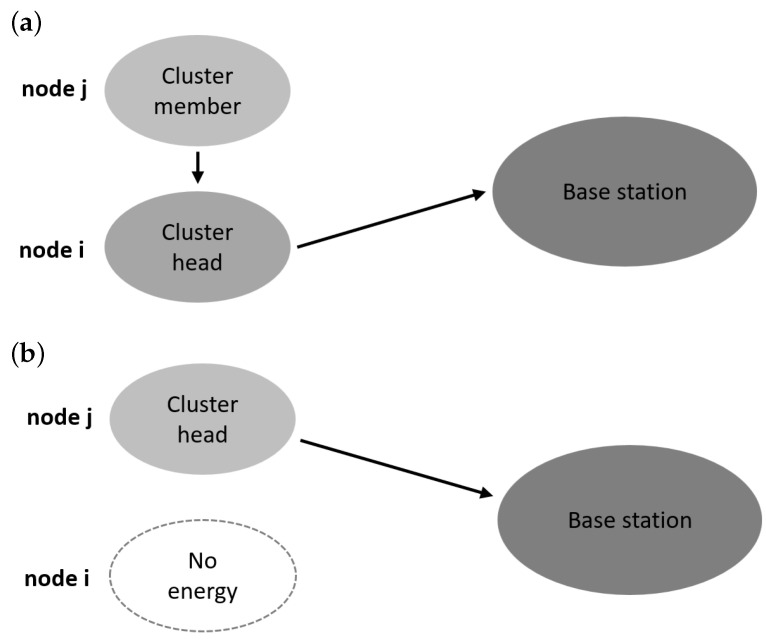
Example of a sensor network with two nodes: (**a**) sensor node *i* initially serves as the cluster head and (**b**) node *j* takes over the role of cluster head after node *i* dies.

To discuss the underlying concepts of our cluster head selection method for extending the network’s LT, we consider a network consisting of two nodes (Figure 2). Let us assume that the symbol *i* denotes the identifier of the sensor node that initially serves as the cluster head, while *j* represents the identifier of the node that takes over the role of cluster head after node *i* dies. Furthermore, we assume that the unit of time corresponds to the duration of a single operational step of the WSN. The time until the energy of node *i* is discharged can be determined as follows:

(2) $$T_{1}=\frac{EI_{i}}{EH}$$

where $EI_{i}$ is the initial energy of node *i* and EH is the energy consumed in a single step (the amount of energy used per unit of time) when the node acts as the cluster head.

After time $T_{1}$, the energy of node *j* is

(3) $$E_{j}\left(T_{1}\right)=EI_{j}-\frac{EI_{i}}{EH}\cdot EM_{j},$$

where $EI_{j}$ is the initial energy of node *j* and $EM_{j}$ is the energy consumed per time step by node *j* when it acts as a cluster member.

After the death of node *i*, node *j* acts as the cluster head until its energy is depleted. The duration of this period can be calculated as follows:

(4) $$T_{2}=\frac{E_{j}\left(T_{1}\right)}{EH}=\frac{EI_{j}}{EH}-\frac{EI_{i}\cdot EM_{j}}{EH^{2}}.$$

As a result, for the case under analysis, the LT can be estimated using the following formula:

(5) $$LT=T_{1}+T_{2}=\frac{EI_{i}+EI_{j}}{EH}-\frac{EI_{i}\cdot EM_{j}}{EH^{2}}.$$

It is important to note that when selecting node *i*, we do not alter the value of the sum $EI_{i}+EI_{j}$, nor do we change the value of EH.

Therefore, to extend the LT, it is essential to minimize the product $EI_{i}\cdot EM_{j}$ by appropriately choosing node *i*:

(6) $$EI_{i}\cdot EM_{j}\to \min.$$

Based on Equation (6), we can conclude that node *i* should be the one with the smallest initial energy and that node *j* should be the one that consumes the least energy when acting as the cluster member. The selection of node *i* directly influences the choice of node *j*. Therefore, by modifying the rule for selecting node *j* as previously formulated, we can assert that node *i* should be the one that consumes the most energy when acting as a cluster member. For example, we can consider a scenario where nodes A and B both start with the same or very similar initial energy levels. However, node A transmits data more frequently due to its sensing conditions or application-specific role, resulting in higher energy consumption when acting as a cluster member. In contrast, node B transmits less frequently, meaning that it consumes less energy as a cluster member. In this scenario, we should prefer to assign the cluster head role to node A.

In the considered WSN, the operations of sensor nodes are organized into rounds. Each round comprises a fixed number of time steps, denoted as *k*. During each round, sensor nodes may transmit data to the cluster head depending on sensed events or data importance. These transmissions occur irregularly (i.e., not at every time step) due to the nature of the sensed phenomena and the need to conserve energy. To model and anticipate the behavior of sensor nodes in future rounds, we define a transmission probability $p_{i}$ for each node *i*, which represents the estimated probability that the node will transmit data to its cluster head during a single time step in the current round. This probability is computed based on the transmission activity observed in the previous round, employing a simple but effective prediction mechanism. For each node (*i*), we evaluate the so-called transmission probability $p_{i}$ as follows:

(7) $$p_{i}=E_{i}/k$$

where $E_{i}$ is the number of time steps when transmission from node *i* was necessary during the previous round and *k* is the total number of time steps in one round.

The amount of energy consumed by the cluster member is proportional to the transmission probability $p_{i}$. Thus, according to the above-discussed cluster head selection rule, node *i* should be the one with the highest transmission probability.

**Figure 3:**
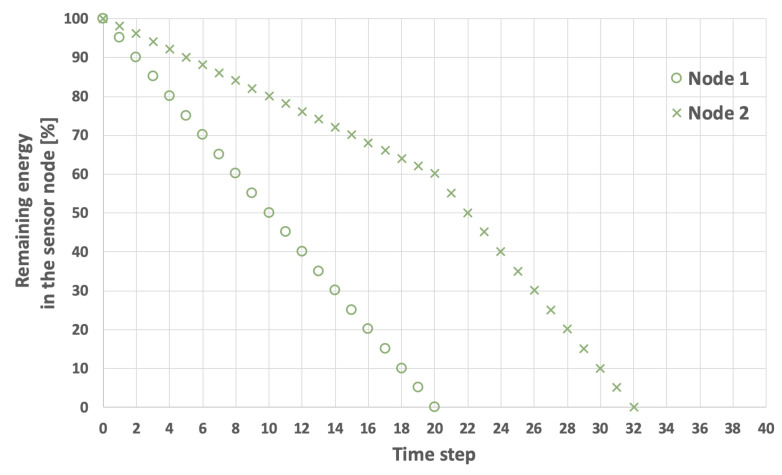
An example of energy consumption by sensor nodes. Node 1 is initially selected as the cluster head, initial energy level is the same for both nodes, LT=32.

**Figure 4:**
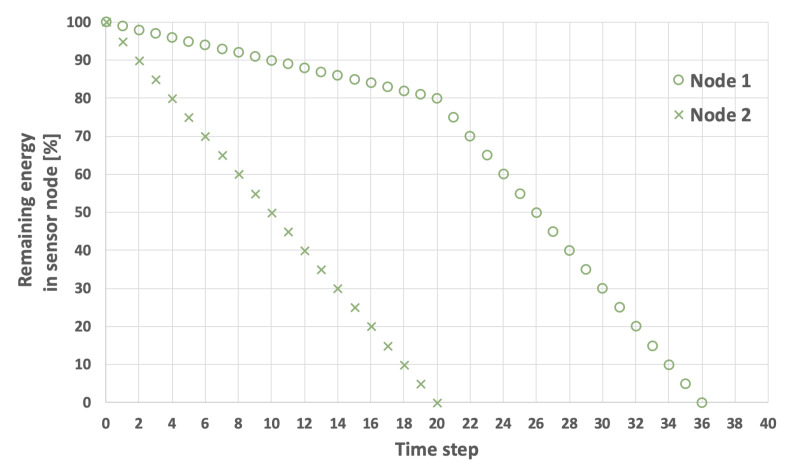
An example of energy consumption by sensor nodes. Node 2 initially selected as the cluster head, initial energy level is the same for both nodes, LT=36.

**Figure 5:**
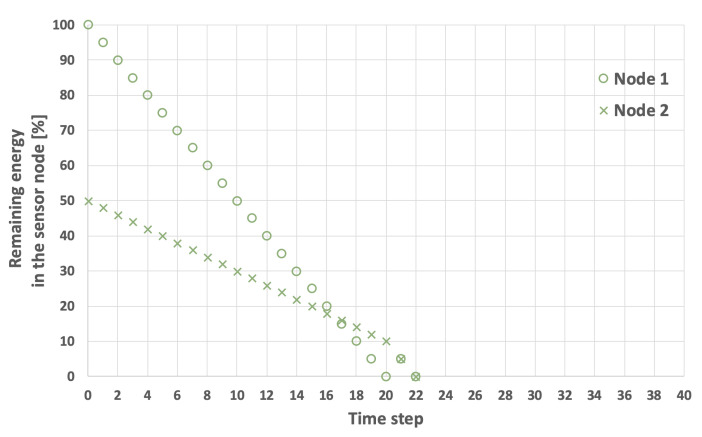
An example of energy consumption by sensor nodes. Node 1 is initially selected as the cluster head, the nodes have different initial energy levels, LT=22.

**Figure 6:**
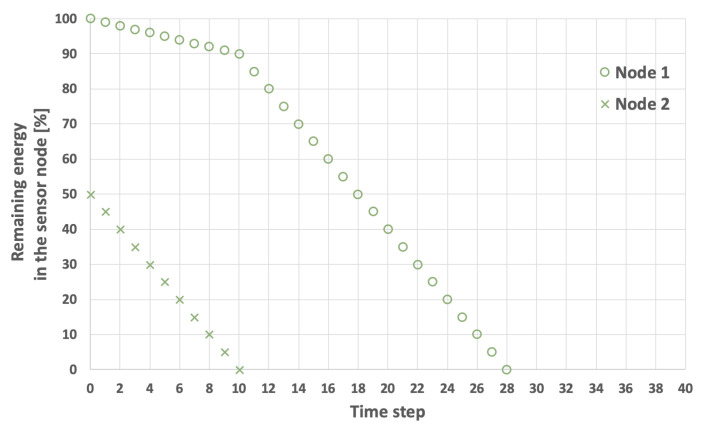
An example of energy consumption by sensor nodes. Node 2 is initially selected as the cluster head, the nodes have different initial energy levels, LT=28.

The problem of extending the network lifetime LT defined by Equation (1) is illustrated in Figure 3, Figure 4, Figure 5 and Figure 6. The examples presented in these figures relate to a network consisting of two nodes. In the scenarios depicted in Figure 3 and Figure 4, nodes 1 and 2 both start with the same initial energy levels of $EI_{1}=100$ and $EI_{2}=100$. Additionally, it is assumed that EH=5 and that the transmission probability for node 1 is lower than for node 2, resulting in $EM_{1}=1$ and $EM_{2}=2$. Figure 3 illustrates the scenario in which node 1 (the node with the lower transmission probability) is selected as node *i*. In this case, the LT is equal to 32 time steps. Conversely, in the example shown in Figure 4, node 2 (which has a higher transmission probability) is chosen as node *i*, extending the LT to 36 time steps.

An example involving sensor nodes with varying initial energy levels is illustrated in Figure 5 and Figure 6. Here, it is assumed that the initial energy level of node 1 is 100, while node 2 has an initial energy level of 50. The other network parameters remain constant. When node 1 is selected as the initial cluster head (i=1), the network lifetime is equal to 22 time steps (Figure 5). Conversely, when the node with the lower initial energy level (node 2) acts as the initial cluster head (i=2), the network lifetime increases to 28 time steps (Figure 6).

The above analysis of a network with two sensor nodes allows us to observe that the selection of the cluster head has a significant impact on the last node’s lifetime. Although real-world clusters typically contain multiple sensor nodes, the fundamental principles that govern efficient cluster head selection can be clearly illustrated in the case with two nodes. The insights gained from these examples are scalable, as the same decision metrics apply when extended to clusters with more nodes. Thus, our algorithm is designed based on the analysis presented above to incorporate scalable parameters and decision criteria, making it applicable and effective in clusters of arbitrary size.

The proposed Algorithm 1 consists of two main steps. In the first step, the nodes with the lowest initial energy are identified as candidates for the role of cluster head. In the second step, the candidate node with the highest transmission probability is selected.

The initial stage of the algorithm involves determining the transmission probabilities $p_{i}$. The transmission probabilities for all sensor nodes are stored in an array *p*. Next, the set of active nodes (those for which $W_{i}=1$) is determined. It is important to note that a sensor node may be inactive ($W_{i}=0$) due to energy depletion or other failures. Candidate nodes are selected from the set of active nodes. A sensor node is identified as a candidate if its initial energy $E_{i}$ does not exceed the minimum initial energy of all nodes by more than 5%. The set of candidate nodes is represented in the pseudocode by the variable *X*. Finally, the candidate node with the highest transmission probability is chosen as the next cluster head.

**Figure 7:**
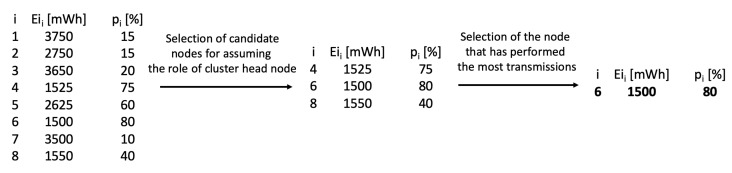
Example of cluster head selection using Algorithm 1.

By considering multiple candidate nodes, the proposed algorithm prevents situations in which minor variations in initial energy levels lead to exclusion of the transmission probability criterion during the cluster head selection process. An example of our algorithm’s operation is illustrated in Figure 7.
**Algorithm 1** The proposed cluster head rotation procedure.**Output**: ID of the node that will serve as the cluster head in the next round.
 **function**
determineClusterHeadNode(*W*, *n*, *E*, *k*, EI)    Initialize array *p*            ▹ transmission probabilities for all nodes    **for** i ← 0 to n−1 **do**    $p_{i}$←$E_{i}$ / *k*    **end for**    *X*← identifiers of nodes *i* for which $W_{i}=1$    *X*← identifiers of nodes i∈X for which $EI_{i}\leq 1.05\cdot \min_{i\in X}EI_{i}$    nextParentNode ← identifier i∈X for which $p_{i}=\max_{i\in X}p_{i}$    **return** nextParentNode
 **end function**


The computational complexity of the proposed cluster head selection algorithm is O(*n* log *n*), where *n* is the number of sensor nodes. While most steps are linear, including computing the transmission probabilities and filtering the nodes, the selection processes for choosing the nodes with high transmission probability and minimum initial energy are performed using sorting, which dominates the overall complexity. Thus, sorting-based minima and maxima computations are the most computationally expensive operations.

## 4. Model Sensor Network

In this section, we present the model sensor network utilized to conduct experimental evaluations of the proposed cluster head rotation method. The primary components of the model include the data transmission algorithms employed by individual nodes and the parameters that characterize the energy consumption of a sensor node at various stages of network operation. The values of these parameters were determined through experiments conducted with the implemented prototype of the sensor network.

The operation of the considered WSN is designed to deliver valuable measurement data from the sensor nodes to the base station. In each operational step of the network, a selected sensor node serves as the cluster head, while the remaining nodes fulfill the roles assigned to cluster members. If a cluster member decides to transmit data to the cluster head during a given step, it activates its radio module to perform the transmission. If not, the transmission is skipped. The cluster head node analyzes measurements from its own sensors and additionally gathers information from the cluster members before forwarding the collected data to the base station.

Each round of the WSN operation consists of an equal number of steps. After the last step of a round, the algorithm selects the node that will act as the cluster head during the next round. Initialization and assignment of node roles are performed during the initial stage of network operation. During the configuration phase, each node is assigned a unique logical address (ID), allowing the identification of individual nodes in the WSN. Each round of WSN operation consists of an equal number of steps. After the final step of each round, a node is selected to serve as the cluster head for the subsequent round.

During the WSN configuration phase, each node is assigned a unique ID, enabling the identification of individual nodes within the network. After the network is activated, the node with the lowest ID begins to transmit synchronization frames, thereby assuming the role of the cluster head. The higher the ID, the longer a node will wait to receive the synchronization frame. Consequently, the remaining nodes are assigned the role of cluster members, either due to their longer listening period for the synchronization frame or their later activation time. Following the initialization process, the sensor nodes enter a sleep mode, the duration of which depends on the objectives of the sensor network and the requirements regarding how frequently measurements must be taken.

The operations performed by the cluster members are outlined in Algorithm 2. When analyzing the provided pseudocode, it is important to note that it pertains to the operation of a node within a single operational time step. During a node’s operation, a periodic increment of the step counter occurs. The next operation involves acquiring data from sensors, after which the node makes a decision regarding data transmission. The instruction labeled “decide whether data should be transmitted” represents a decision-making process that is dependent on the specific application. In practice, this may involve assessing whether the sensed data exceed a predefined threshold, indicate a significant event, or meet specific relevance criteria. In our experimental environment, this decision is modeled using a predefined transmission probability assigned to each node, which reflects the likelihood of the node generating useful data at any given time. This abstraction facilitates the emulation of application-aware or event-driven behavior in which nodes with higher transmission probabilities are more active, resulting in varying energy usage. If the sensor node determines that the data should be sent to the cluster head, then it proceeds with the transmission. To conserve energy, sensor nodes activate the communication module only when data transmission is necessary.

After the node has completed the predefined number of steps, the procedure for selecting the cluster head node is initiated. This process begins with the activation of the communication module, as the cluster member will be waiting for the synchronization frame to be transmitted by the cluster head at this stage. If the node does not proceed to the cluster head node selection procedure during a specific network operation step, then the communication module is deactivated for a defined period (sleeptime).

Algorithm 3 describes the operation of the cluster head. The procedure begins with the increment of the time step counter. The next operation is to activate the communication module and transmit a synchronization frame to all nodes in the cluster. The synchronization frame contains data that are essential for new nodes to join the network, i.e., the current step counter state and the cluster head node identifier. This allows new nodes to join the network at any time during the round.

After transmitting the synchronization frame, the cluster head reads data from its sensors along with the other cluster members, then waits to receive data from the remaining nodes in the cluster. The value of the datareceptiontime parameter is appropriately determined to ensure that the cluster head can collect data from all cluster members. After receiving the data, the transmission counter *E* is updated at each step of the node’s operation. The value of $E_{i}$ is incremented if cluster member *i* has reported its sensor readings to the cluster head at a given time step. Furthermore, for the cluster head (i.e., when *i* represents the ID of the current cluster head), the transmission counter $E_{i}$ is incremented when the cluster head determines that its own sensor readings need to be transmitted to the base station.
**Algorithm 2** Cluster member operation.
   step ← step + 1   read data from sensors   decide whether data should be transmitted   **if** data should be transmitted **then**         turn on communication module         send data to cluster head   **end if**   **if** step = laststep **then**       **if** communication module is off **then**             turn on communication module      **end if**      start cluster head selection procedure   **end if**   turn off communication module (sleeptime)


The next operation involves data aggregation and transmission to the base station. If the node has completed the predefined number of steps, then the round concludes and the procedure for selecting the cluster head node begins. After this procedure is complete, the communication module is set to sleep mode.
**Algorithm 3** Cluster head operation.
    step ← step + 1    turn on communication module    send synchronization frame to all cluster member nodes    read data from sensors    wait for data from cluster member nodes (datareceptiontime)    update transmission count $E_{i}$    aggregate data    send aggregated data to base station    **if** step = laststep **then**          start cluster head node selection procedure    **end if**    turn off communication module (sleeptime)


After completing a specified number of steps in the operation of the sensor nodes (specifically, at the end of each round), the procedure for selecting the cluster head is initiated (Algorithm 4). The course of this procedure depends on the role that the node is currently performing. If the node is acting as the cluster head, then its task is to determine the ID of the node that will assume this role in the next round. In this context, the proposed cluster head rotation approach (Algorithm 1) can be utilized or an alternative state-of-the-art method may be employed. After a new cluster head is selected, a synchronization frame containing the newly assigned cluster head ID is transmitted to all nodes within the cluster. It is essential that all nodes listen to this frame simultaneously in order to prevent situations where only some nodes receive the correct cluster head identifier. Such errors could lead to improper node operation, frequent executions of the initialization algorithm, and negative impacts on the overall lifetime of the sensor network.

The operations performed by the cluster members during the selection of a new cluster head begin with listening for the synchronization frame. This process ends when the frame is received or when a specified timeout occurs, as defined by the variable datareceptiontime. If a node does not receive the synchronization frame, then the initialization procedure is initiated. Conversely, when the synchronization frame is received, the node checks the ID of the cluster head contained within it. If the ID matches that of the node performing this operation, then that node assumes the role of the cluster head in the upcoming round. Otherwise, the node takes on the role of a cluster member, and the cluster head ID is interpreted as the recipient address to which data must be sent in the new round.
**Algorithm 4** Procedure for selecting the cluster head.
    **if** node role = cluster head **then**          run the appropriate method for selecting the new cluster head          send synchronization frame to all nodes in the cluster          turn off communication module (sleeptime)    **else if** node role = cluster member **then**          start listening for the synchronization frame          **repeat**
                wait
      
    **until** received synchronization frame or datareceptiontime has passed           **if** synchronization frame received **then**                 **if** cluster head node ID = own ID **then**                        node role = cluster head node                  **else**                        node role = cluster member node                  **end if**                  turn off communication module (sleeptime)          **else**                start initialization procedure          **end if**    **end if**    step = 0


The procedure for determining the lifetime of the WSN is outlined in Algorithm 5. The process begins with the initialization of all sensor nodes and the assignment of their initial roles. The next step involves executing a single round of sensor network operations, which consists of performing *k* operational steps for the nodes. In each operational step, a designated sensor node acts as the cluster head and consumes energy defined by the variable $S_{\mathrm{CH}}$. The remaining nodes are designated as cluster members. The energy consumption of a cluster member accounts for two scenarios. If, a cluster member transmits data to the cluster head in a particular step, then it consumes an amount of energy, represented by the variable $S_{\mathrm{TRANS}}$; otherwise, no transmission occurs and the node consumes an amount of energy determined by the variable $S_{\mathrm{NOTRANS}}$. Upon completing the round, the cluster head selection procedure is executed. After this procedure is finished, the variable roundcounter is incremented by one. In the subsequent step, the number of active nodes is assessed. If there are no active nodes, the WSN operation terminates, and the algorithm returns the total number of rounds during which the network performed its tasks.
**Algorithm 5** Procedure for determining the lifetime of a sensor network.**Output**: Sensor network lifetime (number of rounds)    **function** determineNetworkLifetime(*W*, *s*, *n*, *k*, $S_{CH}$, $S_{TRANS}$, $S_{NOTRANS}$)          perform node initialization          **while true do**                **for** j←0 to k−1 **do**           ▹ LoRaWAN technology, k=60                      **for** i←0 to n−1 **do**                            **if** role of the *i*-th node = cluster head node **then**                                  perform a cluster member node operation step                                  $s_{i}\leftarrow s_{i}-S_{CH}$                            **end if**                            **if** role of the *i*-th node = cluster member **then**                                  perform a cluster head node operation step                                  **if** *i*-th node transmitted data in the current step **then**                                        $s_{i}\leftarrow s_{i}-S_{TRANS}$                                 **else**                                        $s_{i}\leftarrow s_{i}-S_{NOTRANS}$                                 **end if**                           **end if**                      **end for**                      execute cluster head node selection procedure                **end for**                round counter ← round counter +1                activeNodes ←0                **for** i←0 to n−1 **do**                      **if** $s_{i}>\epsilon$ **then**                            activeNodes ← activeNodes +1                      **end if**                **end for**                **if** activeNodes = 0 **then**                      **break**                **end if**          **end while**          **return** round counter    **end function**


It is important to note that all variables in Algorithms 1–4 are considered local to individual sensor nodes and are not shared between nodes. Each node operates independently based on its own state and sensor readings. However, in Algorithm 5, which describes the global simulation of the entire WSN, all variables used in the pseudocode should be treated as global, meaning that they represent the current state of all nodes in the network and are accessible for evaluating the network lifetime.

To validate the algorithms presented in this section, we implemented and tested them in a prototype WSN. Details of the experimental testbed are described in Section 5. These tests confirmed the feasibility of practically implementing the proposed approach and allowed us to determine the amount of energy consumed by sensor nodes under conditions that closely resemble real-world scenarios. Next, we calibrated our WSN model based on the physical implementation. The calibration process included measurements that enabled us to determine the parameters $S_{\mathrm{CH}}$, $S_{\mathrm{TRANS}}$, and $S_{\mathrm{NOTRANS}}$. Detailed results are presented in the following section.

## 5. Experiments

The experimental testbed was established using LoRaWAN technology [33,34]. A LoRaWAN sensor network can comprise nodes (including both cluster heads and cluster members), gateways (concentrators), a network server (base station), and application servers. In designing a sensor network that employs this technology, it was assumed that the data transmitted by the cluster head could be received by multiple gateways [35]. The software operating on the gateway is responsible for forwarding incoming traffic to the network server. This process can be executed using cellular networks, Ethernet, WiFi, or satellite technology. Subsequently, the network server facilitates the transmission of LoRaWAN messages by relaying data to the primary application servers. In addition to the aforementioned data transmission scheme, direct communication between end nodes is also feasible, which was utilized during our research.

The WSN prototype was constructed using modules based on the Arduino MKR WAN 1310 board (Arduino S.r.l., Monza, Italy), which provides connectivity to the IoT Cloud along with existing LoRaWAN infrastructure such as The Things Network and other boards through direct communication mode. The prototype board comprised three independent components: a 32-bit SAMD21 microcontroller based on the ARM Cortex-M0 core, the Murata wireless communication module, and the ECC508 integrated circuit, which facilitates hardware support for data transmission encryption. Notably, the manufacturer also supplied a power supply stabilization block. In battery-powered systems it is possible to disconnect this block from the rest of the circuit, which positively impacts the reduction of energy consumption by the node.

When the microcontroller and the LoRaWAN radio module are in deep sleep mode, the current consumption is measured at 16.6 μA, which allows for an extended operational time for the sensor node. Given the relatively long initialization time of the LoRaWAN radio module, it was determined that the network operation step would be set to one second, with synchronization conducted at one-minute intervals.

The communication module (Murata CMWX1ZZABZ, Murata Investment Co., Ltd., Shanghai, China) operates in accordance with the LoRaWAN 1.0.x specification, implementing a pure ALOHA-based uncoordinated medium access protocol at the MAC layer and supporting Class A functionality, which is typical for ultra-low-power devices. In LoRaWAN Class A, following each uplink transmission (from the device to the gateway), the device opens two predefined receive windows (RX1 and RX2), during which it may receive a downlink response from the network.

**Figure 8:**
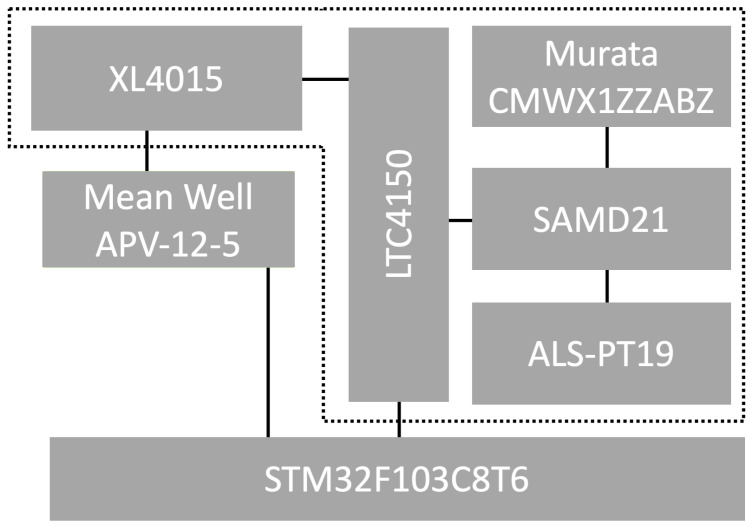
Part of the experimental testbed for a single sensor node.

The algorithms for rotating the cluster head role were implemented on the SAMD21 microcontroller and the STM32 family circuit along with the LTC4150 module, which measures energy consumption for a given network node, as illustrated in Figure 8 (modules integrated into a single sensor node are indicated with a dashed line). Each node was equipped with the ALS-PT19 (Everlight Electronics Co., Ltd., Taipei, Taiwan) analog light sensor. During the research, a Mean Well APV-12-5 (Mean Well Enterprises Co., Ltd., New Taipei City, Taiwan) stabilized power supply and a step-down converter based on the XL4015 (XLSemi Microelectronics Co., Ltd., Shanghai, China) circuit were utilized to reduce the supply voltage to the 3.3 V level required for the prototype board. Energy consumption was measured over a period encompassing 43,200 operational steps for each node.

The energy consumption measurements were conducted using the LTC4150 module [36], which generates a pulse on a dedicated output each time the sensor node consumes 0.1707 mAh. By analyzing the recorded number of pulses generated by the LTC4150 module and the known measurement duration, it was possible to determine the energy consumption per operational step of the sensor node. The energy measurements reflect the raw module usage only. Losses due to power regulation are not included in the reported values. These measurements focused on the energy consumption of the sensor node in the following scenarios: when the node acts as a cluster head ($S_{\mathrm{CH}}$), when it functions as a cluster member and transmits data to the cluster head ($S_{\mathrm{TRANS}}$), and when it operates as a cluster member but does not transmit data to the cluster head ($S_{\mathrm{NOTRANS}}$).

Energy consumption measurements were carried out over a period of six hours for each of the node’s operating scenarios. Based on the conducted studies, it was observed that the master node consumed energy equal to 838 mWh. When the node performed the role of a cluster member without transmitting data to the cluster head, the measurement system recorded an energy consumption of 655 mWh. In situations where the cluster member did not engage in data transmission, it consumed 40 mWh of energy. By dividing the obtained measurement results by the number of steps during which the measurements were taken, the values for the parameters $S_{\mathrm{CH}}$, $S_{\mathrm{TRANS}}$, and $S_{\mathrm{NOTRANS}}$ required for the simulation algorithm were determined. A summary of the energy consumption measurements by the sensor nodes is presented in Table 2.

After determining the parameters that describe energy consumption in the sensor nodes, we used the procedure described in Algorithm 5 to assess the lifetime of the WSN. Algorithm 5 was employed to determine the network lifetime for each method under comparison. The operation “execute cluster head node selection procedure” in Algorithm 5 was implemented separately for each method examined during the experiments. Dedicated implementations were developed for the basic approach, LEACH, EDCR, and the proposed method.

It should be noted that each round in the examined WSN lasts for one minute. The ϵ parameter was set to the $S_{\mathrm{CH}}$ value. The network’s lifetime was determined as the total number of rounds counted from the network’s initialization until the last node ceased to function. For example, if the network operated for 3330 rounds, the resulting network lifetime would be 55 min and 30 s.

For the purpose of the experiments, a WSN consisting of ten nodes was considered. The experiments were conducted with constant and variable transmission probabilities. In the case of time-varying transmission probability, changes occurred every 5 min within 15-min cycles. In the second scenario, the transmission probability remained constant throughout the duration of the experiment, as presented in Table 3. The transmission probabilities were arbitrarily selected to represent a variety of possible node behaviors in WSNs. These values are not derived from real-world measurements but were defined to simulate both static and dynamic transmission scenarios. The data transmission behavior was simulated probabilistically in both constant and time-varying transmission probability scenarios. At each time step of WSN operation, a random number uniformly distributed in the interval [0, 1] was generated independently for each sensor node. A node was assumed to transmit data to the cluster head if the generated number was less than or equal to its current transmission probability. In the time-varying case, the probability thresholds were updated every 5 min according to the pattern shown in Table 3.

For the first series of experiments, it was assumed that each node was equipped with a fully charged battery with a capacity of 2310 mWh.

**Figure 9:**
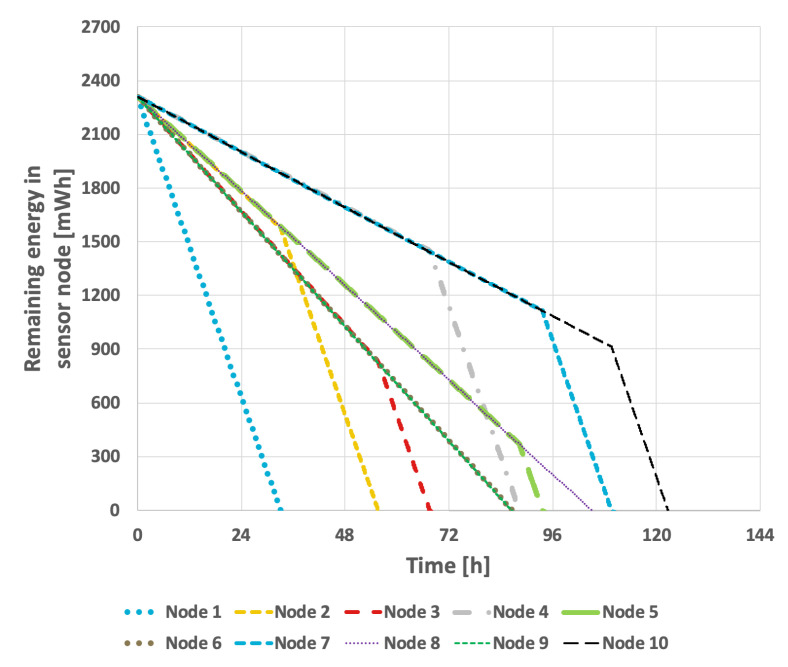
Energy consumption by sensor nodes for the basic approach and the EDCR algorithm (constant data transmission probability).

**Figure 10:**
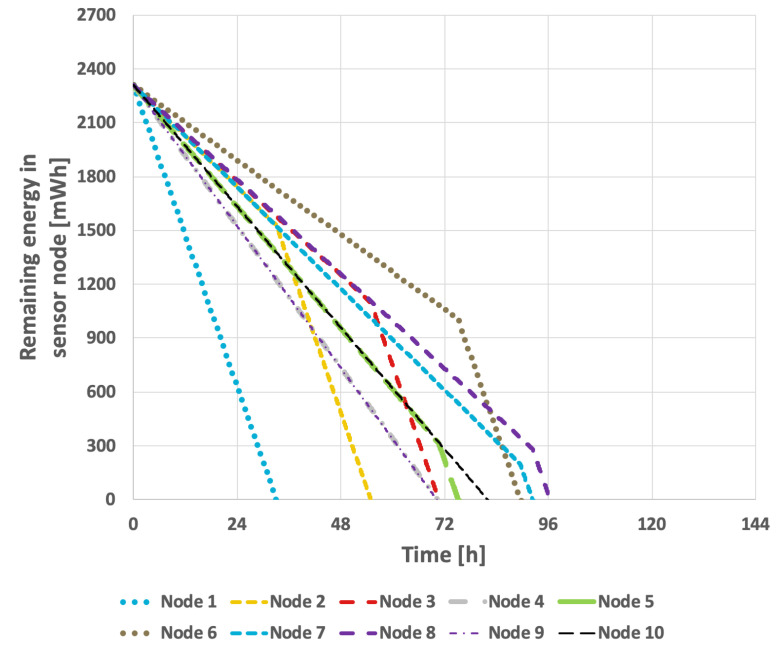
Energy consumption by sensor nodes for the basic approach and the EDCR algorithm (variable transmission probability).

Figure 9 presents the results obtained from the basic approach, in which the node with the lowest identifier is designated as the cluster head. After its battery is depleted, the role is successively transferred to the next active node in ascending order of identifiers. In this scenario, the network lifetime was recorded at 122 h and 39 min. Figure 10 illustrates the situation with a variable transmission probability, resulting in a reduced lifetime of 96 h and 25 min. A significant drawback of this approach is that the assignment of identifiers to individual nodes within the sensor network critically influences the overall lifetime. The network lifetime declines sharply when nodes that transmit the least data are depleted first. This method has considerable limitations, as it is challenging to predict the number of transmissions each node will perform and how to assign their identifiers—which determine the order in which they assume the cluster head role—so as to maximize the network lifetime.

**Figure 11:**
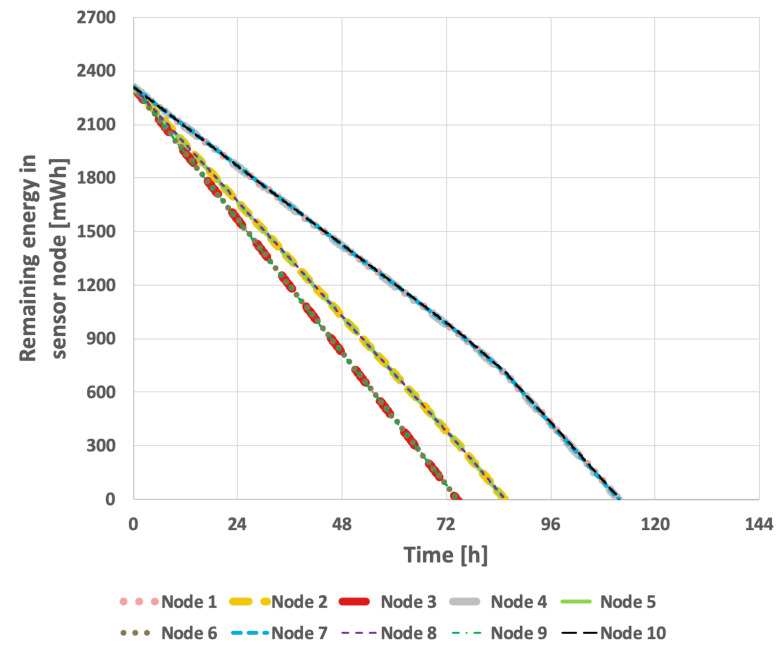
Energy consumption by sensor nodes for the LEACH method (constant transmission probability).

**Figure 12:**
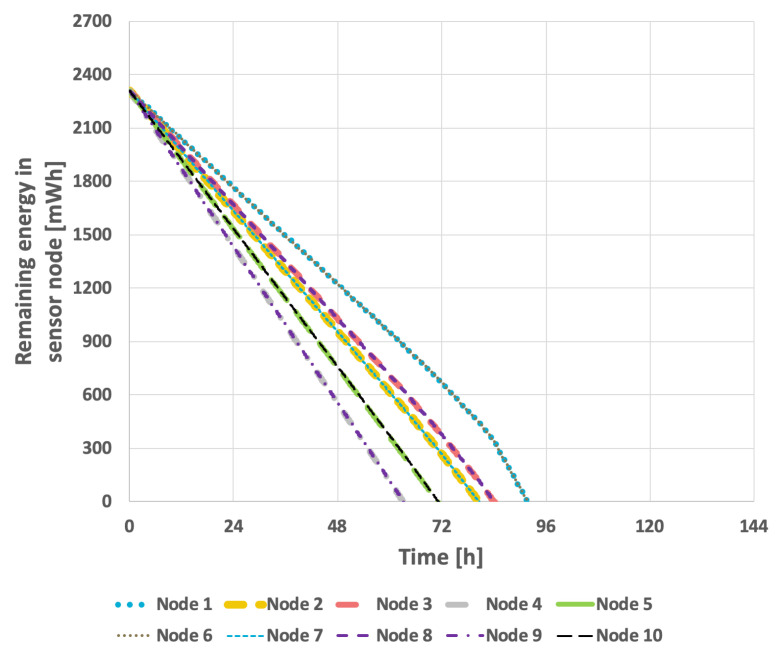
Energy consumption by sensor nodes for the LEACH method (variable transmission probability).

Figure 11 and Figure 12 illustrate the results obtained using the LEACH method. In the case of constant transmission probability, the network lifetime was 111 h and 43 min. In the second scenario, this was reduced to 91 h and 51 min. The conducted studies indicate that the effectiveness of extending the network’s lifetime with this approach is relatively low.

Experiments were conducted to compare the lifetimes achieved using the state-of-the-art methods described in Section 2, which aim to achieve uniform energy consumption among sensor nodes. As anticipated, these methods resulted in a shorter time to the death of the last node compared to the proposed approach, as they were specifically designed to prolong the time until the first node dies. A representative example of the aforementioned state-of-the-art methods is EDCR.

**Figure 13:**
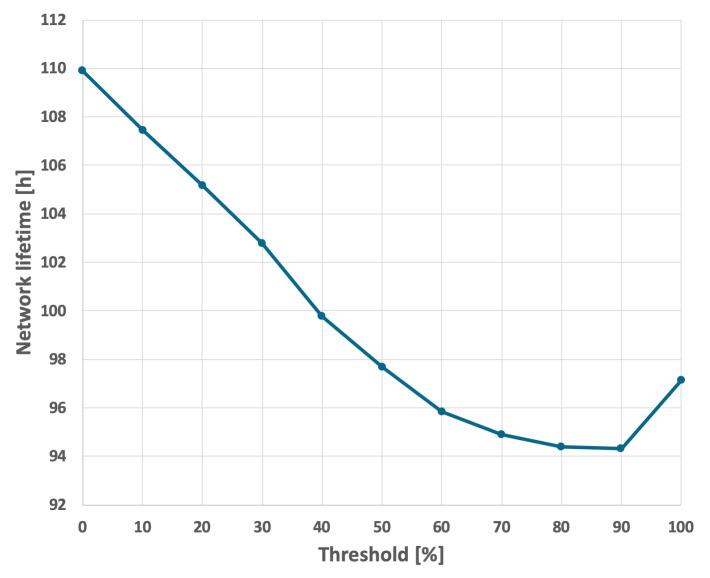
Calibration of the EDCR algorithm (constant data transmission probability).

**Figure 14:**
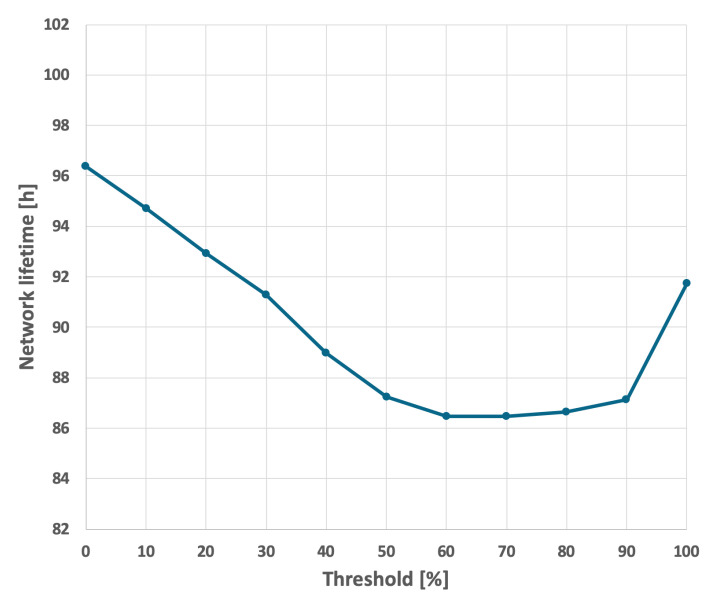
Calibration of the EDCR algorithm (variable data transmission probability).

For the EDCR protocol, the initial step involves determining the energy threshold, after which the algorithm transfers the role of the cluster head to the subsequent node. Following the calibration process for both constant and variable transmission probabilities, an energy threshold of 0% was established, as illustrated in Figure 13 and Figure 14. This indicates that the transfer of the parent role occurs only after the node is completely discharged, effectively reducing the method to the basic approach in which nodes are sequentially discharged based on the identifiers assigned to them during the design phase. As shown in Figure 9 and Figure 10, it can be observed that the network lifetime was 122 h and 45 min in the case of constant transmission probability, whereas for periodic changes in transmission probability the lifetime was 96 h and 21 min.

**Figure 15:**
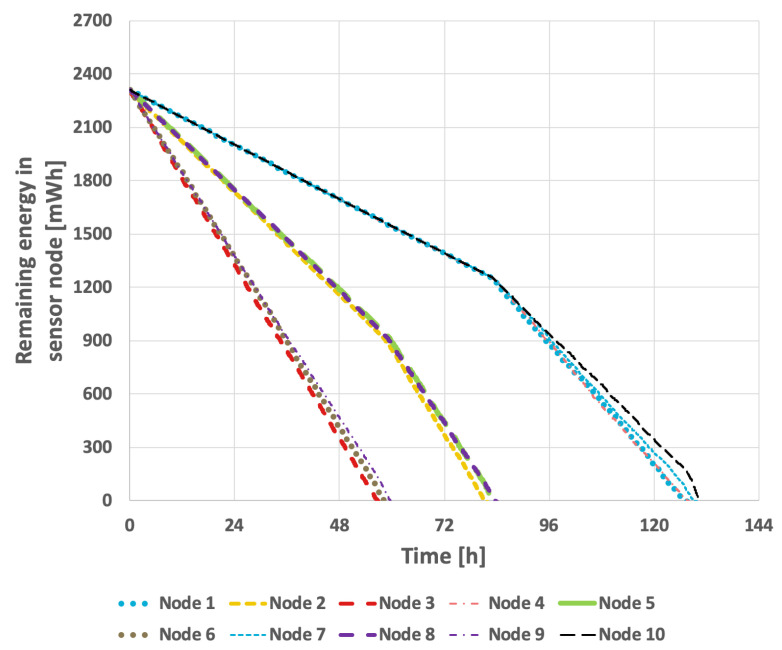
Energy consumption by sensor nodes for proposed method (constant data transmission probability).

**Figure 16:**
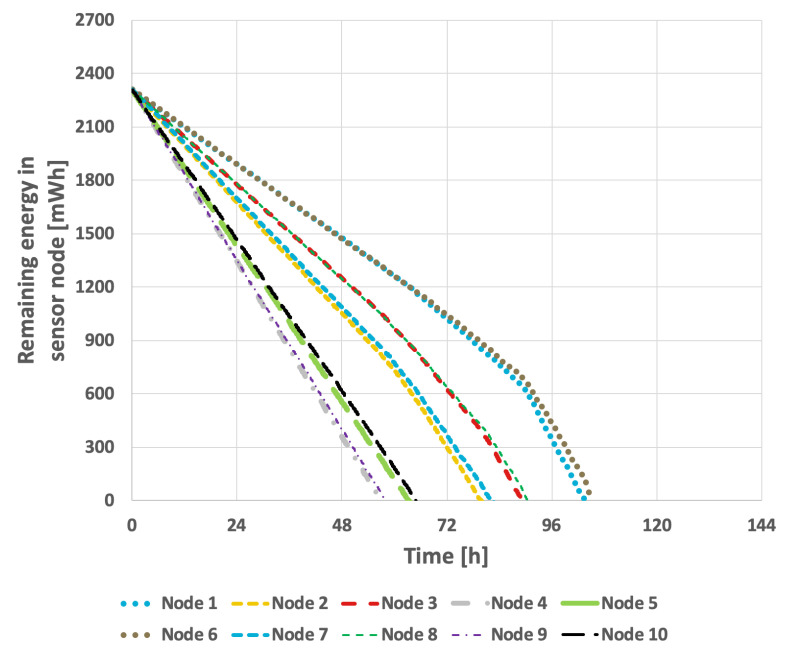
Energy consumption by sensor nodes for proposed method (variable data transmission probability).

The results obtained for the proposed method are presented in Figure 15 and Figure 16. In the scenario where the transmission probability is constant, the proposed method extended the network lifetime to 130 h and 15 min. Because the proposed method accounts for the current transmission probabilities, which are calculated in each round, the response to changes that necessitate selection of a new cluster head occurs much more rapidly. Consequently, the network lifetime in the case of variable transmission probability was also extended, reaching 105 h and 7 min.

**Figure 17:**
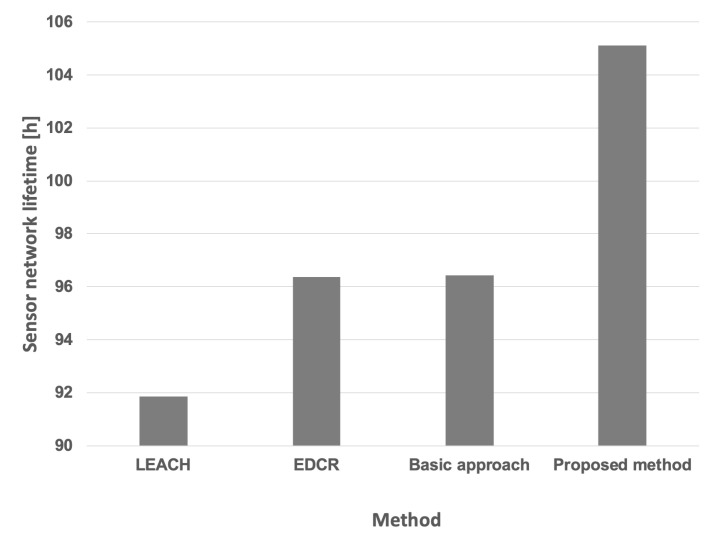
Network lifetime for the compared methods (equal initial energy levels of nodes, variable data transmission probability).

Table 4 presents the aggregated results obtained for both the proposed approach and other available methods, assuming the same initial energy level for all nodes under both constant and variable data transmission probabilities. Furthermore, the Wilcoxon statistical test was conducted to demonstrate the statistical significance of the results, with a significance level of p<0.05. The symbol ↑ indicates that the indicated algorithm is statistically superior to all other algorithms examined in this test. Lifetimes achieved for the variable data transmission probability are also illustrated in Figure 17.

Based on the above results, it can be observed that the longer time to death of the last node in the WSN when using the proposed algorithm is primarily attributed to its ability to handle transmission probabilities in the cluster head selection process. The proposed algorithm selects the node with the highest current transmission probability as the cluster head in each round. This design ensures that nodes which are expected to transmit data more frequently (i.e., that consume energy at a higher rate) are prioritized to perform the cluster head role earlier. Consequently, nodes with lower transmission probabilities (which consume less energy as cluster members) are left to perform the cluster head role at the end of the sequence. The final cluster head retains a higher energy reserve compared to the others; after all other nodes deplete their energy while serving as cluster heads, this last node can continue to operate as the cluster head for longer, extending the network lifetime. In contrast, the compared existing approaches (EDCR and LEACH) aim to equalize energy consumption among nodes, preventing any single node from depleting too early. This strategy tends to distribute energy usage more uniformly, limiting the possibility of preserving a reserve of energy to sustain network operation at later stages.

The next series of experiments aimed to verify the effectiveness of different algorithms in a scenario where only some nodes have fully charged batteries. The initial available energy for individual sensor nodes is presented in Table 5, while the probabilities of data transmission considered during the experiments are provided in Table 3.

**Figure 18:**
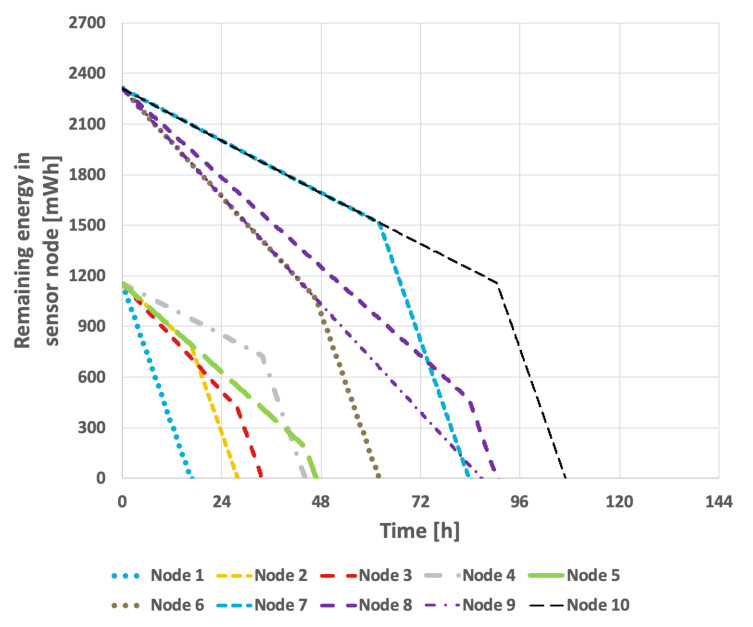
Energy consumption by sensor nodes for the basic approach and the EDCR algorithm (different initial energy levels of nodes, constant data transmission probability).

**Figure 19:**
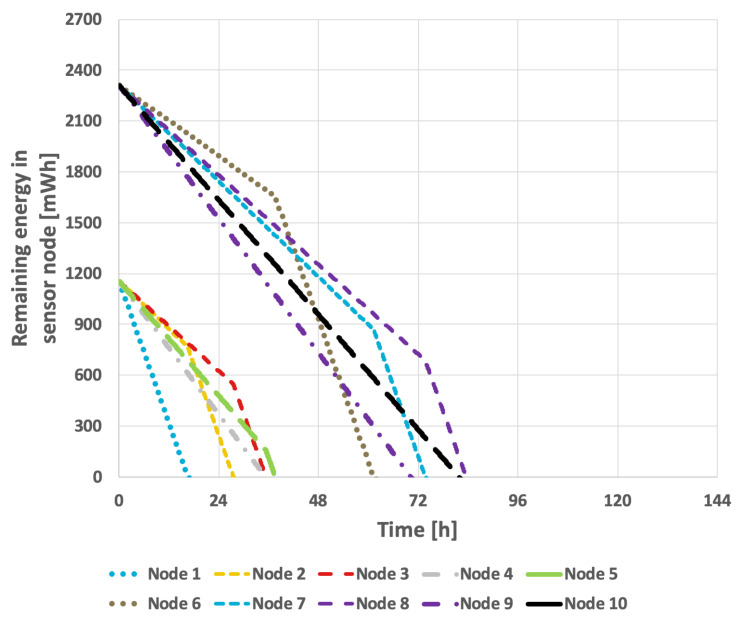
Energy (different initial energy levels of nodes, variable transmission probability).

Figure 18 and Figure 19 present the results obtained for the basic approach, in which the node with the lowest identifier is designated as the cluster head. When its battery is depleted, the role is successively transferred to the next active node in ascending order of identifiers.

Figure 18 illustrates the network lifetime when the transmission probability was held constant, resulting in the lifetime of 107 h. In contrast, when the data transmission probability fluctuated, the recorded network lifetime was 83 h and 33 min, as depicted in Figure 19.

**Figure 20:**
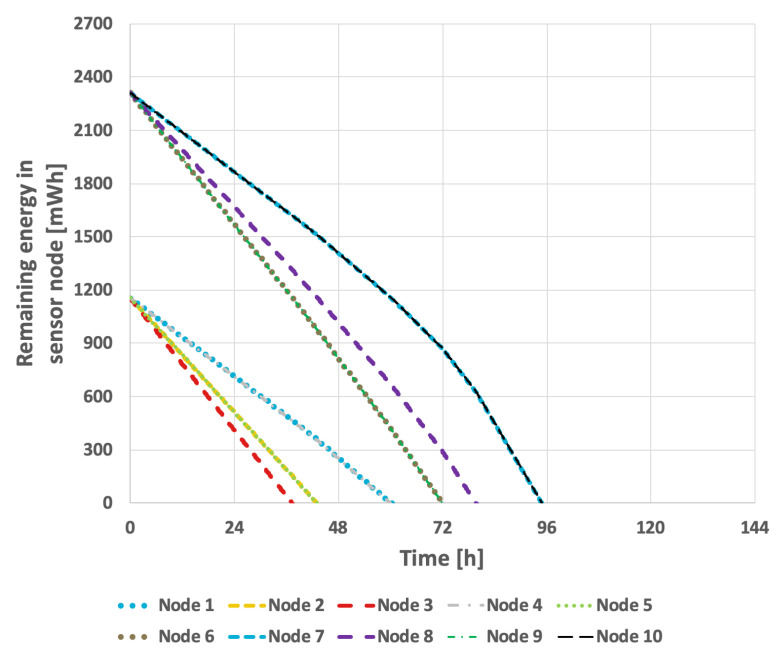
Energy consumption by sensor nodes for the LEACH method (different initial energy levels of nodes, constant transmission probability).

**Figure 21:**
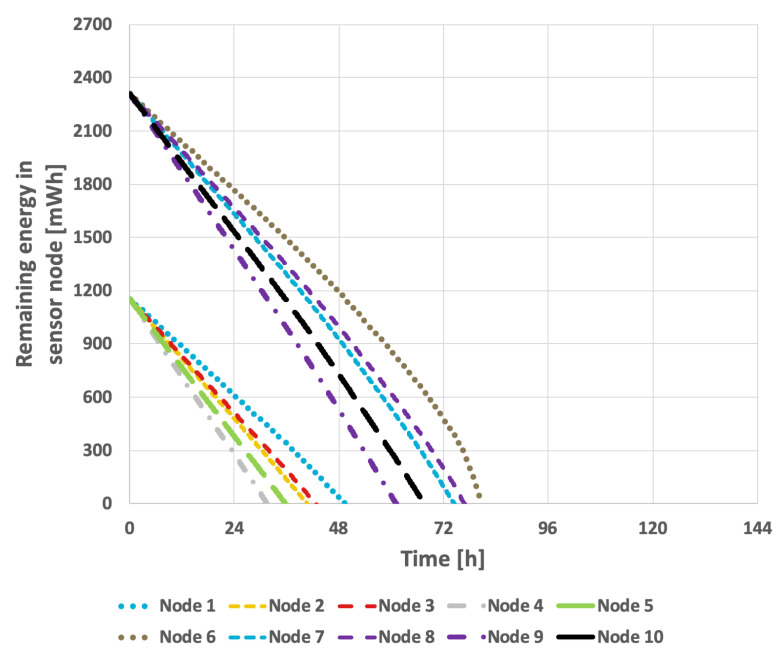
Energy consumption by sensor nodes for the LEACH method (different initial energy levels of nodes, variable transmission probability).

Figure 20 and Figure 21 present the results obtained for the LEACH method. In the scenario where the data transmission probability remained constant, the network lifetime was 94 h and 52 min. When the data transmission probability varied over time, the network lifetime decreased to 80 h and 54 min. These results indicate that the LEACH algorithm is not well-suited for use in a network where individual nodes are equipped with batteries of varying capacities.

**Figure 22:**
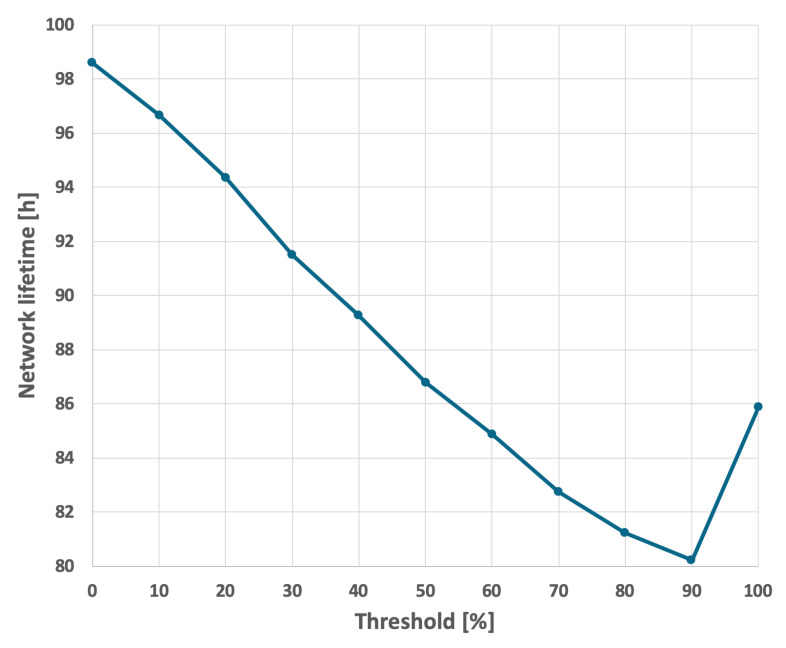
Calibration of the EDCR algorithm (different initial energy levels of nodes, constant data transmission probability).

**Figure 23:**
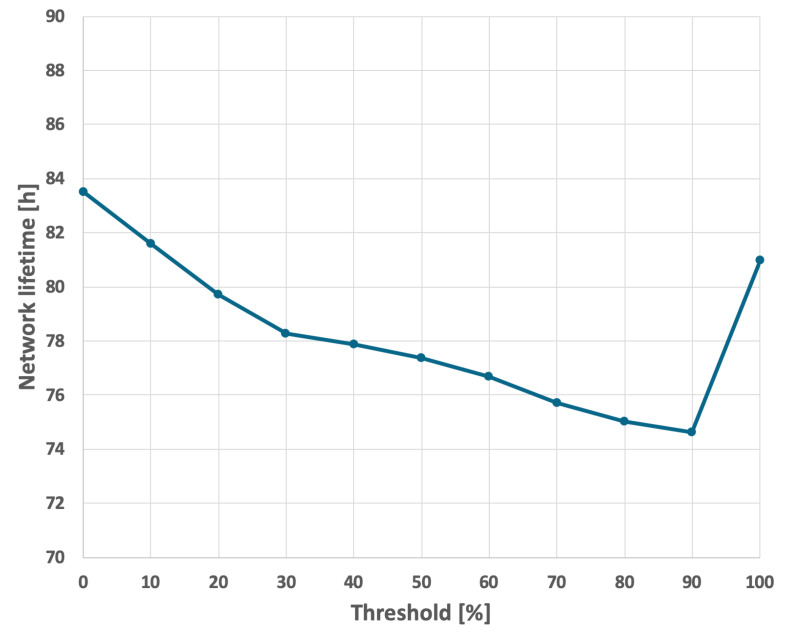
Calibration of the EDCR algorithm (different initial energy levels of nodes, variable data transmission probability).

The results for the EDCR algorithm are presented in Figure 18 and Figure 19. This approach necessitates a calibration process, as illustrated in Figure 22 and Figure 23. Based on these results, it is evident that the longest network lifetime was achieved with a threshold of 0%, indicating that the cluster head node should be replaced only after the current node is completely discharged. This aligns with the basic approach; therefore, the results in Figure 18 and Figure 19 are identical for both methods.

Implementing the EDCR algorithm to extend network lifetime makes the assignment of node identifiers even more critical to the results. This is due to the necessity of considering two key parameters when configuring the network, namely, the number of transmissions recorded by each node and its battery charge level. The network lifetime is comparable to that achieved with the basic approach, reaching 106 h and 57 min for a constant transmission probability and 83 h and 28 min in the second scenario.

**Figure 24:**
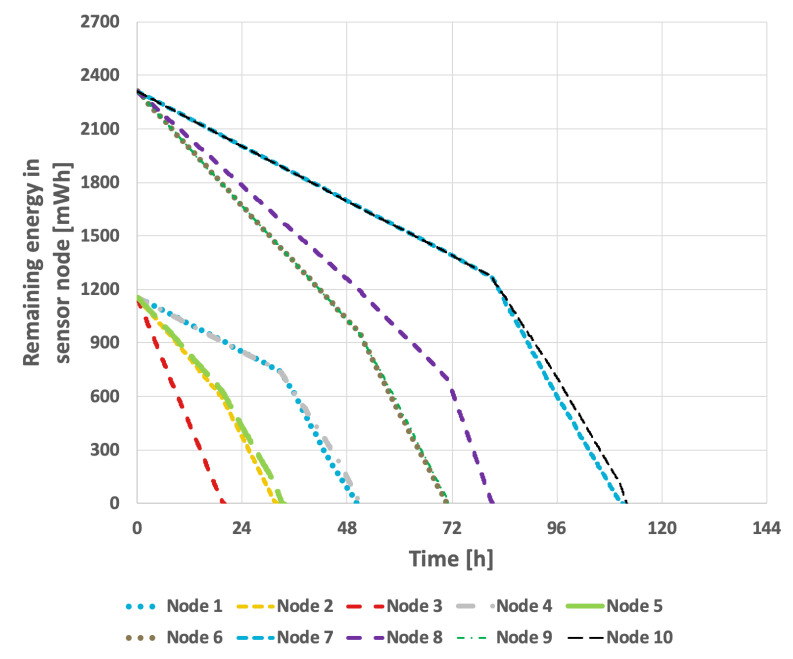
Energy consumption by sensor nodes for the proposed method (different initial energy levels of nodes, constant data transmission probability).

**Figure 25:**
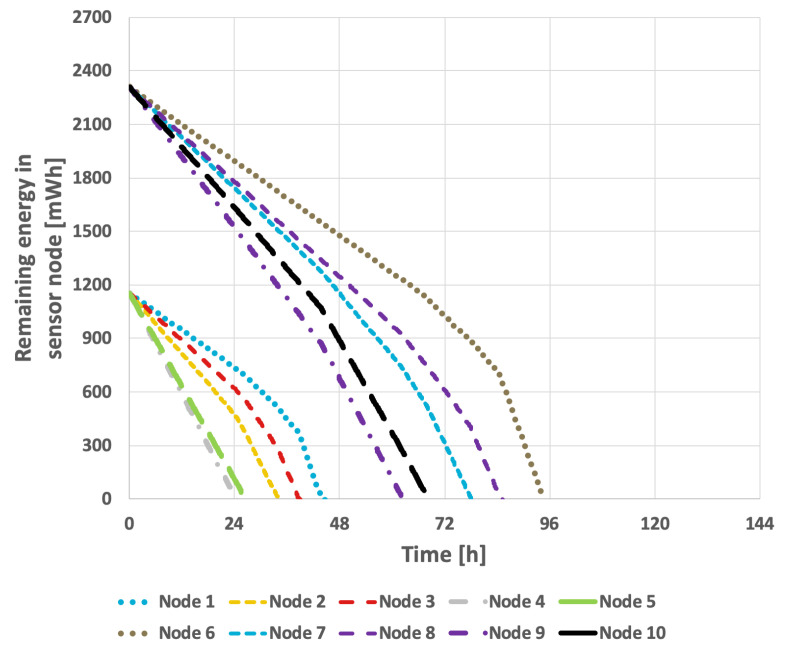
Energy consumption by sensor nodes for the proposed method (different initial energy levels of nodes, variable data transmission probability).

The results obtained when applying the proposed method are presented in Figure 24 and Figure 25. By taking the initial battery charge level into account, the proposed solution effectively extends the network’s lifetime. In the scenario with a constant data transmission probability, the network’s lifetime reached 111 h and 57 min. It is important to note that the proposed solution accommodates situations in which a node transmits data only during selected operational steps. Consequently, in the scenario where the data transmission probability experiences cyclic variations, the network’s lifetime was extended to 94 h and 33 min.

**Figure 26:**
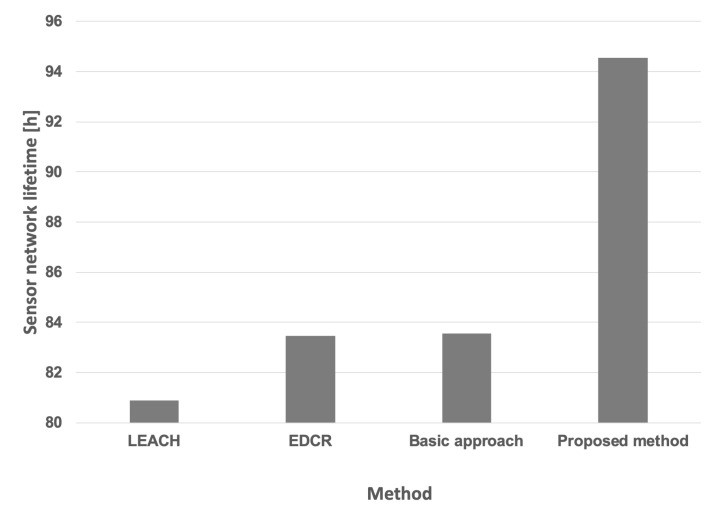
Network lifetime for the compared methods (different initial energy levels of nodes, variable data transmission probability).

Table 6 and Figure 26 present the aggregated results obtained for the proposed approach and the other methods considering different initial energy levels in all nodes for both constant and variable data transmission probabilities. The conducted experiments demonstrate that the lifetime of the prototype sensor network is significantly extended when using our proposed method when compared to the network lifetime achieved using the other algorithms. To confirm the statistical significance of the results, an additional Wilcoxon statistical test was performed, with a significance level of p<0.05.

Based on the results obtained in the second series of experiments where the sensor nodes had different initial energy levels, it was observed that the improvement in extending the time to death of the last node is primarily due to the proposed algorithm selecting the node with the lowest initial energy as the initial cluster head. This decision has a critical impact on the network lifetime. By initially assigning the most energy-constrained node to the cluster head role, the proposed algorithm ensures that this node contributes to network operation while it still has sufficient energy. In other methods, a low-energy node kept in the network as a cluster member during early rounds may expend its energy passively and become depleted before being assigned the cluster head role. The EDCR method prioritizes the selection of nodes with the highest available energy for the cluster head role. Although this approach is effective in balancing energy consumption, it is not well suited for maximizing the time to the last node’s death. By continuously assigning the cluster head role to high-energy nodes, low-energy nodes are left to consume their energy slowly in the cluster member role, and may never be utilized as cluster heads before becoming depleted.

## 6. Conclusions and Future Work

In this paper, we have proposed a novel cluster head selection algorithm for WSNs that extends the time until the last sensor node depletes its energy. The proposed algorithm is based on a formal analysis for the minimal setup that considers network lifetime as a function of energy consumption in sensor nodes. By examining a two-node network scenario, we derive a cluster head selection rule that applies to larger clusters and prioritizes the node with the highest transmission probability and lowest initial energy as the cluster head.

Although the proposed cluster head selection algorithm is initially developed based on detailed analysis of a network with only two sensor nodes, is inherently scalable and is designed for application in clusters with many sensor nodes. The key decision metrics (initial energy and transmission probability) remain valid and critical in larger clusters. The proposed algorithm operates using distributed per-cluster computations. In a WSN that is partitioned into clusters, the cluster head selection process is executed independently within each cluster. This ensures that the algorithm’s complexity does not grow significantly with the overall size of the network. As a result, the proposed algorithm is scalable and can be applied to WSNs with an arbitrary numbers of nodes, provided that the network is appropriately clustered.

Experimental validation of our method using a sensor network prototype demonstrated that the proposed algorithm significantly extends the operational lifetime of the last active node. Our results indicate that existing algorithms which focus on balancing energy depletion among nodes are ineffective in maximizing the time until the last node dies. Instead, our research shows that sensor nodes should be depleted in a specific order that can be determined using the proposed method. By adhering to this depletion sequence, the last sensor node remains operational for a longer duration, effectively extending the useful lifetime of the WSN.

In addition, we implemented an experimental WSN model that accurately reflects real-world WSN behavior. The results obtained with the experimental model confirm that our method consistently achieves longer network lifetimes compared to traditional approaches that do not consider transmission probabilities during cluster head selection.

Based on the experiments conducted in this research, the lifetime of the last sensor node in our prototype WSN was significantly extended when using the proposed method. Our experiments included a comparison between the proposed method and representative existing algorithms from the literature that consider both the time and the available energy of nodes when rotating the role of cluster head.

The results of our experiments demonstrated that algorithms in which the role of the cluster head is transferred after a specific period provide suboptimal utilization of energy resources. This inefficiency arises because the sensor nodes in the WSN consume energy at varying rates. Our research confirms that methods which determine the next cluster head based on available energy levels in order to balance energy consumption among individual nodes are ineffective when the objective is to extend the time until the last node dies.

While most cluster head selection algorithms in the literature are evaluated primarily through simulations, our research distinguishes itself by validating the proposed algorithm using real-world prototypes of sensor nodes based on LoRaWAN technology. This practical evaluation allowed us to capture real environmental influences such as hardware-specific energy consumption patterns and transmission delays. Testing on physical sensor nodes ensures the feasibility and robustness of our algorithm in realistic deployment scenarios.

The outcome of this study also includes recommendations for future research directions concerning cluster head rotation algorithms in WSNs. One potential direction is the development of methods that account for redundant nodes, which could assume the responsibilities of a given node in the event of battery depletion. Another avenue for exploration is the consideration of nodes equipped with various energy sources and the ability to harvest energy from their environment [37,38]. Additionally, the proposed method can be further refined and enhanced by incorporating suitable algorithms for predicting transmission probabilities from individual nodes as well as by mechanisms for managing more complex node hierarchies that involve nodes equipped with different sets of sensors.

## Figures and Tables

**Table 1:** Comparison of LEACH-based protocols.

Protocol	CH Selection Method	Clustering Type	Rotation Mechanism	Advantages	Limitations
LEACH	Random probabilistic	Distributed	Random per round	Local data aggregation	Unbalanced CH distribution
LEACH-C	Centralized (based on energy and location)	Centralized	Recomputed per round	Balanced CH placement	Additional overhead to BS
LEACH-D	Nearest neighbor of current CH (if energy > threshold)	Distributed	Localized CH handover	Smooth transitions	Fails if neighbors lack energy
LEACH-F	Fixed clusters, round-robin CH	Centralized (initial only)	Round-robin	No re-selection needed	Not suited for mobile networks

**Table 2:** Energy consumption per step of a sensor node’s operation in different operational scenarios.

Parameter	Energy Consumption [mWh]
$S_{\mathrm{CH}}$	0.019403
$S_{\mathrm{TRANS}}$	0.015178
$S_{\mathrm{NOTRANS}}$	0.000939

**Table 3:** Transmission probabilities considered during determination of network lifetime.

Sensor Node Identifier									
1	2	3	4	5	6	7	8	9	10
**Constant probability of transmission**									
10%	30%	40%	10%	30%	40%	10%	30%	40%	10%
10%	30%	40%	10%	30%	40%	10%	30%	40%	10%
10%	30%	40%	10%	30%	40%	10%	30%	40%	10%
**Variable probability of transmission (changing every 5 min) **									
30%	30%	30%	40%	70%	30%	30%	30%	40%	70%
10%	20%	20%	70%	10%	10%	20%	20%	70%	10%
20%	50%	40%	50%	50%	20%	50%	40%	50%	50%

**Table 4:** Network lifetime for the compared methods (equal initial energy levels of nodes).

Data Transmission Probability	Method	Network Lifetime
Constant	Basic approach	122 h 39 m
	LEACH	111 h 43 m
	EDCR	122 h 45 m
	Proposed method	130 h 15 m ↑
Variable	Basic approach	96 h 25 m
	LEACH	91 h 51 m
	EDCR	96 h 21 m
	Proposed method	105 h 7 m ↑

**Table 5:** Assumed initial available energy in sensor nodes during the experiments.

Sensor Node Identifier									
1	2	3	4	5	6	7	8	9	10
**Initial available energy in sensor node [mWh]**									
1155	1155	1155	1155	2310	2310	2310	2310	2310	2310

**Table 6:** Network lifetime for the compared methods (different initial energy levels of nodes).

Data Transmission Probability	Method	Network Lifetime
Constant	Basic approach	107 h
	LEACH	94 h 52 m
	EDCR	106 h 57 m
	Proposed method	111 h 57 m ↑
Variable	Basic approach	83 h 33 m
	LEACH	80 h 54 m
	EDCR	83 h 28 m
	Proposed method	94 h 33 m ↑

## Data Availability

Data are contained within the article.

## References

[1] Kandris D., Anastasiadis E. (2024). Advanced wireless sensor networks: Applications, challenges and research trends. Electronics.

[2] Carsancakli M.F., Al Imran M.A., Yildiz H.U., Kara A., Tavli B. (2022). Reliability of linear WSNs: A complementary overview and analysis of impact of cascaded failures on network lifetime. Ad Hoc Netw..

[3] Bouguera T., Diouris J.F., Chaillout J.J., Jaouadi R., Andrieux G. (2018). Energy consumption model for sensor nodes based on LoRa and LoRaWAN. Sensors.

[4] Casals L., Mir B., Vidal R., Gomez C. (2017). Modeling the energy performance of LoRaWAN. Sensors.

[5] Liando J.C., Gamage A., Tengourtius A.W., Li M. (2019). Known and unknown facts of LoRa: Experiences from a large-scale measurement study. ACM Trans. Sens. Netw. (TOSN).

[6] Pukrongta N., Kumkhet B. The relation of LoRaWAN efficiency with energy consumption of sensor node. Proceedings of the 2019 International Conference on Power, Energy and Innovations (ICPEI).

[7] Rawat P., Chauhan S. (2023). A survey on clustering protocols in wireless sensor network: Taxonomy, comparison, and future scope. J. Ambient. Intell. Humaniz. Comput..

[8] Shafique T., Soliman A.H., Amjad A., Uden L., Roberts D.M. (2024). Node Role Selection and Rotation Scheme for Energy Efficiency in Multi-Level IoT-Based Heterogeneous Wireless Sensor Networks (HWSNs). Sensors.

[9] Rawat P., Chauhan S. (2021). Clustering protocols in wireless sensor network: A survey, classification, issues, and future directions. Comput. Sci. Rev..

[10] Lewandowski M., Płaczek B. (2019). An event-aware cluster-head rotation algorithm for extending lifetime of wireless sensor network with smart nodes. Sensors.

[11] Mostarda L., Navarra A., De Leone R. (2023). Optimal vs rotation heuristics in the role of cluster-head for routing in IoT constrained devices. Internet Things.

[12] Hassan A., Anter A., Kayed M. (2021). A survey on extending the lifetime for wireless sensor networks in real-time applications. Int. J. Wirel. Inf. Netw..

[13] Lata S., Mehfuz S., Urooj S., Alrowais F. (2020). Fuzzy clustering algorithm for enhancing reliability and network lifetime of wireless sensor networks. IEEE Access.

[14] Prasad D., Hassan A., Verma D.K., Sarangi P., Singh S. Disaster management system using wireless sensor network: A review. Proceedings of the 2021 International Conference on Computational Intelligence and Computing Applications (ICCICA).

[15] Yao C., Yang Y., Yin K., Yang J. (2022). Traffic anomaly detection in wireless sensor networks based on principal component analysis and deep convolution neural network. IEEE Access.

[16] Shahraki A., Taherkordi A., Haugen Ø., Eliassen F. (2020). Clustering objectives in wireless sensor networks: A survey and research direction analysis. Comput. Netw..

[17] Gamwarige S., Kulasekere E. An algorithm for energy driven cluster head rotation in a distributed wireless sensor network. Proceedings of the International Conference on Information and Automation.

[18] Gamwarige S., Kulasekere C. Application of the EDCR algorithm in a cluster based multi-hop wireless sensor network. Proceedings of the 2006 International Symposium on Communications and Information Technologies.

[19] Qubbaj N., Taleb A.A., Salameh W. Review on LEACH protocol. Proceedings of the 2020 11th International Conference on Information and Communication Systems (ICICS).

[20] Heinzelman W.R., Chandrakasan A., Balakrishnan H. Energy-efficient communication protocol for wireless microsensor networks. Proceedings of the 33rd Annual Hawaii International Conference on System Sciences.

[21] Daanoune I., Abdennaceur B., Ballouk A. (2021). A comprehensive survey on LEACH-based clustering routing protocols in Wireless Sensor Networks. Ad Hoc Netw..

[22] Mishra P., Alaria S., Dangi P. (2021). Design and comparison of LEACH and improved centralized LEACH in wireless sensor network. Int. J. Recent Innov. Trends Comput. Commun..

[23] Liu D., Liang C., Mo H., Chen X., Kong D., Chen P. (2024). LEACH-D: A low-energy, low-delay data transmission method for industrial internet of things wireless sensors. Internet Things Cyber-Phys. Syst..

[24] Ihsan A., Saghar K., Fatima T., Hasan O. (2019). Formal comparison of LEACH and its extensions. Comput. Stand. Interfaces.

[25] Ullah Z. (2020). A survey on hybrid, energy efficient and distributed (HEED) based energy efficient clustering protocols for wireless sensor networks. Wirel. Pers. Commun..

[26] Dergaoui O., Baddi Y., Hasbi A. Introduce the CH Role Rotation Mechanism in the Multilayered Deterministic WSN Clustering to Achieve Long-Term Load Balancing. Proceedings of the International Conference on Signal Processing and Information Communications.

[27] Mittal N., Singh U. (2015). Distance-based residual energy-efficient stable election protocol for WSNs. Arab. J. Sci. Eng..

[28] Naranjo P.G.V., Shojafar M., Mostafaei H., Pooranian Z., Baccarelli E. (2017). P-SEP: A prolong stable election routing algorithm for energy-limited heterogeneous fog-supported wireless sensor networks. J. Supercomput..

[29] Kamel S.E., Ghanem T.F., Amin K.M., Abdelkader H. Efficient energy management technique for WSNs. Proceedings of the 12th International Conference on Computer Engineering and Systems (ICCES).

[30] Ababneh A.A., Al-Zboun E. (2016). EDAC: A Novel Energy-Aware Clustering Algorithm for Wireless Sensor Networks. Int. J. Adv. Comput. Sci. Appl..

[31] Alkalbani A.S., Mantoro T., Degala S. Energy-Distance Aware Clustering Scheme (E-DACS) for Wireless Sensor Networks. Proceedings of the 12th International Conference on Computing and Information Technology (IC2IT).

[32] Selvi G.V., Anbarasan A.B., Murthy B.A., Prabavathy S. (2023). An Application Oriented Integrated Unequal Clustering Algorithm for Wireless Sensor Network. Underwater Vehicle Control and Communication Systems Based on Machine Learning Techniques.

[33] Almuhaya M.A., Jabbar W.A., Sulaiman N., Abdulmalek S. (2022). A survey on Lorawan technology: Recent trends, opportunities, simulation tools and future directions. Electronics.

[34] Tong S., Wang J., Yang J., Liu Y., Zhang J. Citywide lora network deployment and operation: Measurements, analysis, and implications. Proceedings of the 21st ACM Conference on Embedded Networked Sensor Systems.

[35] Ndukwe C., Iqbal T., Liang X., Khan J., Aghenta L.O. (2020). LoRa-based communication system for data transfer in microgrids. AIMS Electron. Electr. Eng..

[36] Shafiq Z., Egger W. Study of charging strategies of lithium batteries and their effect on the batteries technologies. Proceedings of the 2022 IEEE 13th Annual Information Technology, Electronics and Mobile Communication Conference (IEMCON).

[37] Singh J., Kaur R., Singh D. (2021). Energy harvesting in wireless sensor networks: A taxonomic survey. Int. J. Energy Res..

[38] Sharma P., Singh A.K. (2023). A survey on RF energy harvesting techniques for lifetime enhancement of wireless sensor networks. Sustain. Comput. Inform. Syst..

